# A Distinct Arabidopsis Latent Virus 1 Isolate Was Found in Wild *Brassica hirta* Plants and Bees, Suggesting the Potential Involvement of Pollinators in Virus Spread

**DOI:** 10.3390/plants13050671

**Published:** 2024-02-28

**Authors:** Victoria Reingold, Avi Eliyahu, Neta Luria, Diana Leibman, Noa Sela, Oded Lachman, Elisheva Smith, Yael Mandelik, Asaf Sadeh, Aviv Dombrovsky

**Affiliations:** 1 Department of Plant Pathology and Weed Research, ARO Volcani Center, 68 HaMaccabim Road, P.O. Box 15159, Rishon LeZion 7528809, Israel; vickire@gmail.com (V.R.); neta.luria8@gmail.com (N.L.); diana@volcani.agri.gov.il (D.L.); odlachman@gmail.com (O.L.); elishevasmith@gmail.com (E.S.); 2 Department of Entomology, The Robert H. Smith Faculty of Agriculture, Food and Environment, The Hebrew University of Jerusalem, Rehovot 7610001, Israel; avi.eliyahu9@gmail.com (A.E.); yael.mandelik@mail.huji.ac.il (Y.M.); 3 Department of Natural Resources, Newe Ya’ar Research Center, Agricultural Research Organization, P.O. Box 1021, Ramat Yishay 3009500, Israel; asafsa@volcani.agri.gov.il; 4 The Advanced School for Environmental Studies, The Hebrew University of Jerusalem, Jerusalem 9190401, Israel; 5 Bioinformatics Unit, ARO Volcani Center, 68 HaMaccabim Road, P.O. Box 15159, Rishon LeZion 7528809, Israel; noa@volcani.agri.gov.il

**Keywords:** *Apis mellifera*, *Andrena*, *Eucera*, *Hylaeus*, ArLV1

## Abstract

During our search for aphid-pathogenic viruses, a comovirus was isolated from wild asymptomatic *Brassica hirta* (white mustard) plants harboring a dense population of *Brevicoryne brassicae* aphids. The transmission-electron-microscopy visualization of purified virions revealed icosahedral particles. The virus was mechanically transmitted to plants belonging to *Brassicaceae*, *Solanaceae*, *Amaranthaceae,* and *Fabaceae* families, showing unique ringspot symptoms only on *B. rapa* var. *perviridis* plants. The complete viral genome, comprised of two RNA segments, was sequenced. RNA1 and RNA2 contained 5921 and 3457 nucleotides, respectively, excluding the 3′ terminal poly-adenylated tails. RNA1 and RNA2 each had one open-reading frame encoding a polyprotein of 1850 and 1050 amino acids, respectively. The deduced amino acids at the Pro-Pol region, delineated between a conserved CG motif of 3C-like proteinase and a GDD motif of RNA-dependent RNA polymerase, shared a 96.5% and 90% identity with the newly identified *Apis mellifera*-associated comovirus and Arabidopsis latent virus 1 (ArLV1), respectively. Because ArLV1 was identified early in 2018, the *B. hirta* comovirus was designated as ArLV1-IL-Bh. A high-throughput-sequencing-analyses of the extracted RNA from managed honeybees and three abundant wild bee genera, mining bees, long-horned bees, and masked bees, sampled while co-foraging in a Mediterranean ecosystem, allowed the assembly of ArLV1-IL-Bh, suggesting pollinators’ involvement in comovirus spread in weeds.

## 1. Introduction

The *Comovirus* genus belongs to the *Comovirinae* subfamily in the *Secoviridae* family, which contains a wide variety of members [1]. Comoviruses have a narrow host range that belongs to the *Leguminosae* family, showing characteristic mosaic and mottling symptoms. Viruses belonging to this genus are non-enveloped with icosahedral morphology containing a bipartite positive sense RNA genome [2]. The polyprotein of RNA1 and the small polyprotein of RNA2 are processed into five and three cleavage products, respectively [3,4]. A 3C-like proteinase encoded by RNA1 has unique cleavage sites for polyprotein processing. RNA1 is necessary for viral replication and includes a protease cofactor, a helicase, a viral protein-genome-linked (VPg), a 3C-like proteinase, and an RNA-dependent RNA polymerase (RdRp). The N-terminal protease cofactor of RNA1 assists in processing the RNA2 polyprotein [5]. RNA2 encodes the movement protein and large and small capsid proteins required for viral movement [6]. Comoviruses are transmitted by beetles [7] and mechanical means [2]. A recent study on Arabidopsis latent virus 1 (ArLV1), a comovirus infecting *Arabidopsis thaliana* plants of the *Brassicaceae* family, has shown a highly efficient seed transmission of the virus [8].

Mechanically transmitted viruses are sometimes transmitted by non-host vectors, primarily via mechanical adherence. Bee colonies were considered vectors of plant viruses [9,10,11,12,13,14,15], and metagenomics studies of bee populations were found to be beneficial in the identification of plant viruses in various ecosystems [16,17,18,19,20,21]. The ability to share viruses between managed and wild bees [22,23] may lead to a wide plant virus spread within a specific geographic region [18]. For our study of plant virus spread in wild vegetation, we have sampled the managed honeybees (*Apis mellifera*) and three abundant wild bee genera, mining bees (*Andrena*)*,* long-horned bees (*Eucera*), and masked bees (*Hylaeus*), while co-foraging in a Mediterranean shrub land in central Israel. These four bee genera differ in their nesting, diet, pollen-collecting organs, sociality, body size, and seasonal activity [24]. The honeybee *A. mellifera* is a generalist forager, and compared to most of the wild bees in our system, they are long-distance foragers and are active during most of the year in the examined region. They live in large colonies with a high level of nest-mate interactions. Unlike *A. mellifera*, some mining bees (*Andrena*)*,* long-horned bees (*Eucera*), and masked bees (*Hylaeus*) have narrower diets [25,26,27]; they are short-distance foragers [28,29] and active for short periods limited to a few weeks per year. In the current study of plant virus spread, we have recently found that all the sampled genera harbored a comovirus, which showed similarity with a previously identified comovirus found in asymptomatic *Brassica hirta* (white mustard) plants in Mediterranean shrubland ecosystems in central Israel. The virus found in mustard showed a high similarity to the recently described latent ArLV1. This study emphasizes the likely involvement of wild and managed bees in the spread and persistence of plant viruses in wild vegetation, allowing a possible spread to adjacent economically important or research-essential crops. 

## 2. Materials and Methods

### 2.1. Wild Plant Collections 

During the spring of 2011, asymptomatic *B. hirta* plants, heavily infested with the cabbage aphid (*Brevicoryne brassicae*), were collected from a no-till farming area for virus identification and characterization. The collected *B. hirta* plants, found to harbor a comovirus, were used as source material to establish a mustard comovirus culture. During the winter of 2022, asymptomatic *B. hirta* plants were collected from fields near commercial hives, and the presence of the mustard comovirus was confirmed by RT-PCR using the primer sets F10-R8 and F15-R13 for RNA1 and RNA2, respectively (Table 1) (see below).

### 2.2. Viral Particle Purification and Transmission Electron Microscopy Visualization

Virus purification was conducted as described previously [30] with some modifications. Briefly, 100 g of mustard leaves were homogenized in 200 mL of 0.067 M sodium phosphate buffer (pH 7.2) and 50 mL of 0.1 M ascorbic acid. The homogenate was centrifuged at 9700× *g* for 10 min at 4 °C. The separated supernatant was mixed with 10% chloroform and shaken for 10 min at 4 °C, followed by a pH adjustment to 5.3. The mixture was centrifuged at 200,000× *g* for 2 h in a fixed-angle (Beckman Ti 35) rotor. The pellets were suspended in a cold 0.01 M sodium phosphate buffer (pH 7.0). Transmission electron microscopy (TEM) was used to identify the purified viral particles. For TEM visualization, a purified virion sample (3.5 μL) was applied onto 300 mesh carbon-coated copper TEM grids for 30 s. Excess fluids were blotted, and after a wash with distilled water, the grids were stained with 2% uranyl acetate and visualized using a Tecnai G^2^, FEI-Philips (Philips, Eindhoven, The Netherlands).

### 2.3. Viral RNA Extractions from Purified Virions 

Viral RNA extractions from viral particles were conducted as described previously [31,32]. In brief, purified virions were subjected to RNase-free DNase I digestion (Promega; Madison, WI, USA) (1 h at 37 °C) followed by Proteinase K treatment (1 h at 37 °C). The viral RNA was purified using acid-phenol chloroform (Ambion/Applied Biosystems; Austin, TX, USA). Viral RNA precipitation was conducted overnight at −20 °C in the presence of glycogen (Fermentas-Thermo Fisher Scientific; Burlington, ON, Canada), 0.1 M sodium acetate, and isopropanol. The obtained viral RNA was then washed twice with 75% ethanol, air-dried for 10 min, and suspended in 40 µL of 0.01 M Tris-borate EDTA (TBE) buffer.

### 2.4. Double-Stranded (ds) cDNA Synthesis

Viral RNA samples from purified virions served as a template for cDNA synthesis using random hexamer and oligo dT_17_VN primers with a Maxima reverse transcriptase (Fermentas). The cDNA served as a template for the second strand synthesis using the Universal RiboClone cDNA Synthesis System (Promega, Madison, WI, USA), according to the manufacturer’s instructions. The ds-cDNA was purified using the Zymoclean kit (Zymo Research, Irvine, CA, USA), and the obtained double-stranded fragments were cloned as a library into a commercial pUC19/*Sma*I (Fermentas). The library was then transformed into DH5α competent cells, and insert-positive colonies were cultured in LB media, including antibiotics. The plasmids were extracted using a plasmid extraction kit (Bioneer, Daejeon, Republic of Korea) and sequenced by Sanger sequencing (HyLabs, Rehovot, Israel).

Based on the obtained sequences, the primer pairs were designed (Table 1) and used for RT-PCR to obtain the unidentified genome segments. The 5′ end of the genome was identified using the RACE strategy on cDNA derived from viral RNA extractions, while the 3’ end was sequenced using the oligo dT_17_VN primer.

### 2.5. Viral RNA Extractions from Plants 

The Viral RNA extraction kit (Bioneer, Daejeon, Republic of Korea) was used to extract viral RNA from plant leaves. A sample of 0.5 g of plant tissue was ground in the presence of the supplied RNA extraction buffer, and RNA was extracted according to the manufacturer’s instructions.

### 2.6. RT-PCR Amplifications

Primers were designed based on sequences obtained from the ds-cDNA sequencing to amplify the viral genome. The cDNA served as a template for PCR using JMR polymerase (JMR, Kent, UK) or Advantage 2 Polymerase Mix (Clontech-Takara Bio, Mountain View, CA, USA) and specific primer sets (Table 1). Amplicons were separated on a 1% Agarose gel (HyLabs, Rehovot, Israel), extracted with a Zymoclean Gel DNA recovery kit (Zymo Research, Irvine, CA, USA), and Sanger sequenced (HyLabs). The primer sets F10, R8, and F15, R13 (Table 1) were used for the detection of the mustard comovirus RNA1 and RNA2, respectively. For the detection of Actin, the primer set was as follows: F 5′ ATGCCAACACTGTCCTTTCTGG 3′ and R 5′ GACCCACCAATCCATACGGA 3′.

### 2.7. Virus Host Range Analyses 

The host range was studied on a broad range of plants by sap-mechanical inoculations. Extracted sap from infected mustard leaves, prepared in a 0.01 M sodium phosphate buffer (pH 7.0), was gently rubbed on plant leaves in the presence of carborundum dust. Symptoms were visualized from 14 to 21 days post-inoculation (dpi), and the viral infection was confirmed by RT-PCR using the primer set F8-R5 for RNA1 (Table 1) followed by Sanger sequencing of the amplicons. The tested plants were as follows: *Brassica perviridis* (mustard); *B. rapa* (turnip); *Raphanus sativus* (radish); *B. oleracea* (cabbage); *Nicotiana benthamiana* (benth); *N. glutinosa* (Peruvian tobacco); *Vicia faba* (broad-bean); *Vigna unguiculata* (black-eyed pea); *Chenopodium amaranticolor* (Lambs’ quarters); *C. murale* (nettle-leaved goosefoot); *C. quinoa* (quinoa); *Datura stramonium* (Jimsonweed); *Gomphrena globosa* (globe amaranth); and *Erucaria hispanica* (Spanish pink mustard).

### 2.8. Viral Genome Assembly

Nucleotide sequences obtained from the ds-cDNA library were analyzed using the NCBI database and Basic Local Alignment Search Tool (BLAST, NIH, Bethesda, MD, USA). Genome sequence assembly was generated by DNAMAN (Lynnon BioSoft; Montreal, QC, Canada) and SnapGene (GSL Biotech LLC, Boston, MA), and the reference genomes of the turnip ringspot virus (TuRSV) (accession numbers FJ712026 and FJ712027) [33], radish mosaic virus (RaMV) (accession numbers AB295643 and AB295644), and ArLV1 (accession numbers MH899120 and MH899121). The deduced amino acid sequence was obtained using the ORFfinder from NCBI and SnapGene software version 6.0.4.

### 2.9. Characterization of the Viral Coat Protein 

The molecular weight (MW) of the viral coat protein (CP) subunits was estimated by sodium dodecyl sulfate-polyacrylamide gel electrophoresis (SDS-PAGE). The purified virion preparation was fractionated using a 12% polyacrylamide gel containing 0.4% SDS, as described by Laemmli [34]. The resulting protein bands were visualized by staining the gel with Coomassie Brilliant Blue (Sigma-Aldrich, Burlington, MA, USA). 

### 2.10. Bee Sampling

Bee sampling was conducted between February and May 2021 at five shrubland sites in the Judean foothills, a Mediterranean agroecosystem in central Israel (Table S1). Each site was sampled 5–7 times, at 7–10 day intervals. Sampling included two sets of observations on bees’ flower visits, 30 min each, during peak bee activity hours. Subsequently, 15 individuals from the most abundant bee genera were netted. These included managed honeybees (*A. mellifera*) in all sampling days (*n* = 540), as well as solitary mining bees (*Andrena* spp.; *n* = 376), long-horned bees (*Eucera* spp.; *n* = 208), and masked bees (*Hylaeus* spp.; *n* = 109) (Table S1). The captured bees were immobilized on ice, identified at the genus level, and subsequently kept on dry ice and stored in a −80 °C freezer until processed.

### 2.11. Local Flora Characterization

At the beginning of every sampling day, the local flora available for forage was recorded by counting flowering units for every species from ten 1-square-meter quadrates that were located on two transects in the sites.

### 2.12. Viral RNA Extractions from Bees

Total RNA was extracted from every bee individually using the standard phenol and guanidinium isothiocyanate (TriReagent, Cincinnata, OH, USA) protocol. Briefly, each bee was homogenized in 400 µL of BioTri (BioLab Ltd., Jerusalem, Israel), followed by 10 min incubation. A total of 80 µL of chloroform was added to each tube, followed by vortex, 5 min incubation, and 15 min centrifugation. The upper phase was mixed in a ratio of 1:1 (*v*:*v*) with 9 M LiCl in isopropanol, followed by 1 h incubation at −20 °C for precipitation. Following an 8 min centrifugation, the pellet was washed with 75% ethanol, and elution was performed with 40 µL DDW. The RNA from all the bees was pooled according to genus and enriched for viral RNA using the Viral RNA Extraction kit (Bioneer, Daejeon, Republic of Korea) according to the manufacturer’s instructions. The presence of the mustard comovirus was tested by RT-PCR followed by Sanger sequencing as detailed above.

### 2.13. High Throughput Sequencing (HTS) Analyses

In order to diagnose viruses that are both poly-adenylated and non-poly-adenylated, the RNA pools from the four bee genera were in vitro poly-adenylated and sequenced using Illumina Hiseq2500 (50 cycles) platform (Technion Genome Center, Haifa, Israel). Low-quality sequences were filtered and trimmed using Trimmomatic version 0.39 [35]. Clean reads were scanned for matched viral sequences using VirusDetect software version 1.7 [36]. For mapping the reads, VirusDetect software (version 1.7) employed a pipeline that combined de novo assembly using Velvet software version 1.1.07 [37] with mapping to plant viral references from Genbank utilizing the Burrows-Wheeler Alignment tool (BWA) [38]. In parallel, we also used trinity assembler version v2.13.2 [39]; the assembled contigs were then searched for plant virus sequences using diamond Blastx [40] against the NCBI non-redundant protein database. The depth coverage of the viral contigs was calculated using Bowtie2 alignment [41] and Samtools version 1.7 [42].

### 2.14. Statistical Analysis

To determine the tendency of bees from the dominant genera to forage on brassica plants, we calculated, for every sampling day, the difference between the proportional abundance of the brassica plants in the floral community and the proportion of visits to brassica plants by each bee genus. We included in this analysis only days in which bees from the examined genera visited brassica flowers. Thus, the sample size was in accordance with the number of those days. Due to the non-normal distribution of proportional data, we used the Wilcoxon signed-rank test to compare these values with zero. A significant difference for a genus indicates its tendency to visit or avoid brassica plants relative to their proportional abundance. The masked bees were omitted from this analysis because their activity on brassica plants was too low to allow a meaningful inference. All statistical analyses were performed in R version 4.3.1.

### 2.15. A Phylogenetic Tree Analysis

A phylogenetic tree analysis was performed based on the *Comovirinae* subfamily Pro-Pol deduced amino acid region delineated between the conserved CG motif of the 3C-like proteinase and the GDD motif of the RdRp. Sequences were aligned by multiple sequence alignment using Muscle. The grapevine fabavirus (GFabV) (accession number KX241482) served as an out-group. The tree was constructed using MEGA software version 6 based on the maximum likelihood method with the parameter of 1000 bootstraps.

## 3. Results

### 3.1. A Comovirus Was Identified in Asymptomatic Wild B. hirta Plants 

**Figure 1:**
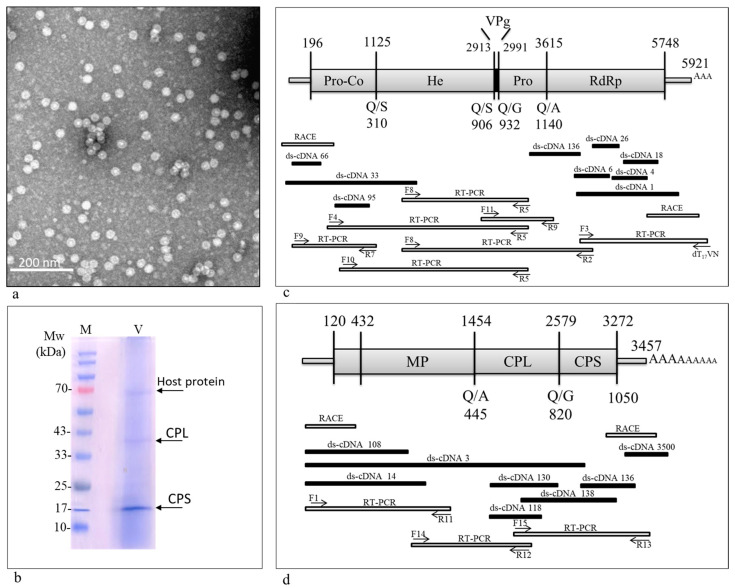
Transmission electron microscopy visualization and genome organization of a comovirus identified in *B. hirta* plants harboring a dense aphid population. (**a**) A representative micrograph showing icosahedral particles. (**b**) A Coomassie brilliant blue stained gel showing predicted ~20 kDa and ~40 kDa CPs. M, molecular size marker; V, virions. (**c**,**d**) Sequencing and genome organization of the mustard comovirus comprised of (**c**) RNA1 and (**d**) RNA2 genome segments.

Wild *B. hirta* plants harboring a dense population of *B. brassicae* aphids were analyzed for viral infection. The TEM visualizations of virions prepared from the plants showed icosahedral particles (Figure 1a). SDS-PAGE analysis of the virion preparation showed two potential CPs of ~20 kDa and ~40 kDa, characteristic of the *Comovirus* genus (Figure 1b). In parallel, several molecular methods were carried out to characterize the entire viral genome sequence. The ds-cDNA procedure was conducted on virion RNA extractions to identify the viral genus. The obtained sequences within the viral genome shared a high similarity with the *Comovirus* genus, which comprises two RNA molecules, RNA1 and RNA2 [43]. The ds-cDNA sequences were aligned and mapped to RNA1 and RNA2 molecules using the reference viruses TuRSV (accession numbers FJ712026 and FJ712027) and RaMV (accession numbers AB295643 and AB295644) (Figure 1c,d). Based on the obtained sequences, primer pairs were designed and used for RT-PCR to attain the unidentified genome segments (Table 1). The 5′ end of the genome was identified using the RACE strategy on cDNA derived from the viral RNA extractions, while the 3’ end was sequenced using the oligo dT_17_VN primer (Figure 1c,d).

### 3.2. Genome Organization

The assembled genome was analyzed using ORFfinder (NCBI) to find the open reading frames (ORFs) and to annotate putative proteins. The ORFs were then identified using the SnapGene software version 6.0.4 and aligned with ArLV1 (accession numbers MH899120 and MH899121). Similar to other comoviruses [2], the mustard isolate showed one ORF within the RNA1, encoding five proteins: a Pro-co, a helicase, a viral protein genome-linked (VPg), a 3C-like proteinase, and an RdRp (i.e., Pol) (Figure 1c). The RNA2 is also comprised of one ORF encoding three known proteins: a movement protein (MP), a large capsid protein (CPL), and a small capsid protein (CPS) (Figure 1d). 

The nucleotide sequence of RNA1, comprised of 5921 nucleotides excluding the polyA tail, showed an 85% similarity with the newly identified *Apis mellifera*-associated comovirus (AmCV) (A) and (B) (accession numbers OP972917 and OP972918), and a 76% similarity with ArLV1 (accession number MH899120). The nucleotide sequence of RNA2, comprised of 3457 nucleotides excluding the polyA tail, showed an 84% and 83% similarity with AmCV (A) and (B), respectively (accession numbers OP972919 and OP972920), but no similarity was found with ArLV1 (accession number MH899121). The deduced amino acid sequence at the Pro-Pol region, encompassing the domain between the conserved CG motif of the proteinase and the conserved GDD motif of the polymerase, shared a 96.5% and 90.3% identity with AmCV and ArLV1, respectively. The deduced amino acid sequence of the CPs shared a 93% and 82% identity with AmCV and ArLV1, respectively. As ArLV1 was already submitted to the GenBank database in 2018, and according to the International Committee on Taxonomy of Viruses (ICTV), the species demarcation criteria for the conserved Pro-Pol region and the CPs were 80% and 75% identical, respectively [2]; the Israeli *B. hirta* isolate was designated as ArLV1-IL-Bh and was deposited to GenBank (accession numbers OR840696 and OR840697). 

### 3.3. 3C-like Proteinase Cleavage Sites and Conserved Motifs in ArLV1-IL-Bh

The putative 3C-like proteinase cleavage sites were in RNA1 at the Pro-Co/Hel domain ^308^VAQ/SGP^313^, at the Hel/VPg domain ^904^VGQ/SRK^909^, at the VPg/Pro domain ^930^WAQ/GTM^935^, and at the Pro/Pol domain ^1138^VVQ^/^AQC^1143^. In RNA2, the putative cleavage sites were at the MP/LCP domain ^443^YGQ/ASV^448^ and at the LCP/SCP domain ^818^EAQ/GVR^823^.

In RNA1 at the Pro-Co region, the conserved amino acids ^124^F, ^151^W, and ^180^E [44] were identified. At the helicase region, the nucleoside triphosphate binding motif had the conserved amino acids ^479^**G**KSRV**GKT**^486^ and the conserved DD preceded by hydrophobic amino acids ^526^ILI**DD**^530^ [45]. The VPg had the conserved amino acids at positions ^909^K, ^914^D, ^918^Y, ^922^N, and ^927^R [46]. The 3C-like cysteine proteinase showed the catalytic cysteine at the ^1100^**CG** motif [47,48]. The RdRp showed the conserved amino acids ^1423^**DYS**S**FDG**LLSK^1433^ and ^1484^**SG**FPL**T**VIC**NS**^1494^, and a GDD with the preceding hydrophobic residue ^1531^Y**GDD**^1534^ and ^1584^**FLKR**^1587^ [49]. In RNA2, the conserved amino acids identified at the MP region were ^69^P, ^129^G, and ^153^D (counting from the second initiation codon at nt 432: GAAATGG, which is placed in a favorable context compared to the first initiation codon at nt 120: ACTATGT) [2,50].

### 3.4. A Host Range Analysis

**Figure 2:**
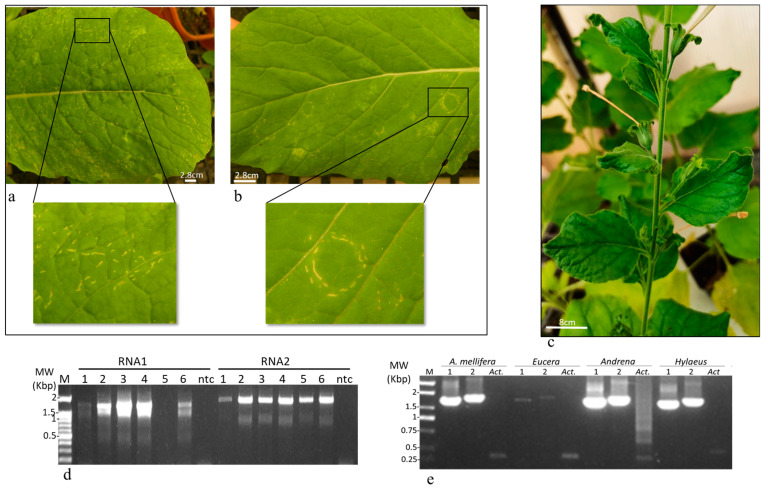
Disease symptoms of ArLV1-IL-Bh. (**a**,**b**) Disease progression symptoms on *B. perviridis* plants infected by ArLV1-IL-Bh. (**a**) Stripes of mild necrotic symptoms. (**b**) A ringspot phenotype. (**c**) Disease symptoms on *N. benthamiana* plants showing mosaic and leaf distortion. (**d**) RT-PCR detecting RNA1 and RNA2 of ArLV1-IL-Bh using primer pairs F10-R8 and F15-R13, respectively (Table 1) in *B. hirta* and *Erucaria hispanica* plants grown in the wild adjacent to domesticated and wild bee populations. 1–3, 5, 6, geographic collection regions of *B. hirta*: 1, Luzit; 2, Galon; 3, Lachish; 5, Tarum; 6, Agur; 4, Lachish- *Erucaria hispanica* plants. (**e**) RT-PCR detecting RNA1 and RNA2 of ArLV1-IL-Bh in bee samples. M, molecular size marker; Act, Actin; ntc, non-template control.

The field-collected asymptomatic *B. hirta* plants served as a source of virus inoculum. The virus was easily transmitted to test plants by mechanical sap inoculation. A broad host range that included members of four plant families was analyzed by RT-PCR (Table 2). Like the wild *B. hirta* plants, most plants that were positive for ArLV1-IL-Bh in RT-PCR analysis were asymptomatic. Unique symptoms of ringspot were observed on *B. perviridis* plants (Figure 2a,b), whereas infected *N. benthamiana* plants showed the classic comovirus symptoms of mosaic and leaf distortions (Figure 2c). 

### 3.5. ArLV1-IL-Bh Identified in Managed and Wild Bee Populations

*B. hirta* (white mustard) and other *Brassicaceae* family species are indigenous to the Israeli wild vegetation, attracting a wide range of insect pests and pollinators such as wild bees and honeybees. Recently collected *B. hirta* plants infected with ArLV1-IL-Bh (Figure 2d) were found in several locations in proximity to managed *A. mellifera* bees and wild bee populations, which were sampled and subjected to RNA extractions and RT-PCR tests for the presence of ArLV1-IL-Bh and HTS analyses (Figure 2e, Table 3). The managed *A. mellifera* bees revealed, by HTS and de novo assembly followed by BLAST analyses, the presence of an *A. mellifera* comovirus isolate, which showed 99–100% sequence identity with the RNA1 of ArLV1-IL-Bh and a contig of 3378 nucleotides showing a 99.8% sequence identity with RNA2 of ArLV1-IL-Bh (Table 4). The Israeli *A. mellifera* comovirus showed a ~90% similarity with each RNA1 of ArLV1 and Zymoseptoria comovirus A and a 96% similarity with RNA1 of AmCV. The large contig covering RNA2 showed an ~80% similarity with each RNA2 of ArLV1 and Zymoseptoria comovirus A and a 92% similarity with RNA2 of AmCV. We, therefore, nominated the Israeli *A. mellifera* comovirus as ArLV1-IL-Am and deposited it to GenBank (accession numbers OR840694 and OR840695).

**Figure 3:**
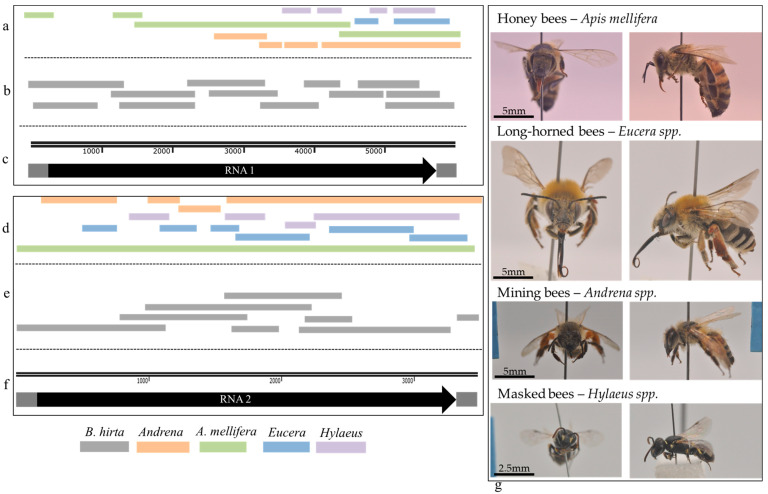
A graphical genome presentation of the ArLV1-IL-Bh, sequence assembly, and alignment with the four bee species’ identified comovirus contigs. (**a**–**c**) RNA1 and (**d**–**f**) RNA2. (**a**,**d**) represent alignments of HTS de novo assembled contigs from wild and managed bees on the genome of ArLV1-IL-Bh. (**b**,**e**) represent the genome amplicons generated from *B. hitra*-infected plants sequenced by Sanger. (**c**,**f**) represent the genome size of both RNA particles, where the dark gray color represents the 3’ and 5’ UTRs, and the black color represents the polyprotein coding region. (**g**) studied bee genera frontal and lateral view (left and right, respectively).

Following the alignments of HTS de novo assembled contigs, the ArLV1-IL-Bh large genome segments were also identified in the wild bee populations, mining bees (*Andrena)*, long-horned bees *(Eucera)* and masked bees (*Hylaeus)*, and showed the highest similarity with the bee comovirus contigs compared to ArLV1, Zymoseptoria comovirus A, and AmCV (Table 4, Figure 3). 

**Figure 4:**
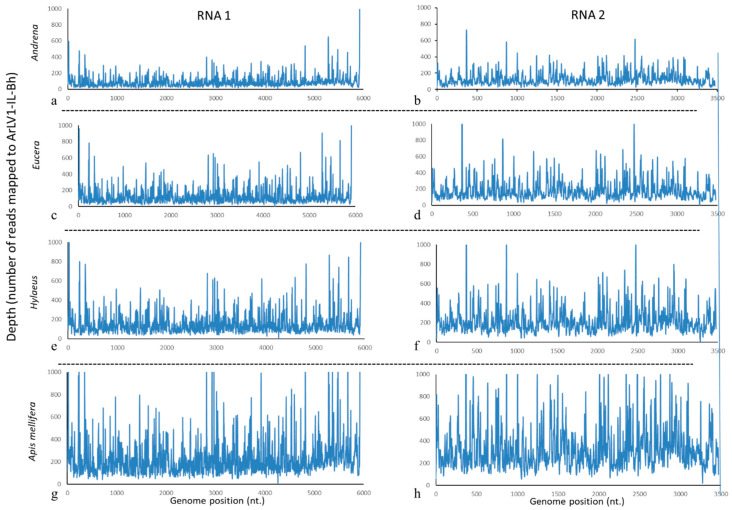
Assembly of bees’ HTS reads on ArLV1-IL-Bh reference RNA1 and RNA2 genome segments. (**a**–**h**) HTS Depth and coverage of ArLV1-IL-Bh genome segments of RNA1 and RNA2. (**a**,**b**), *Andrena*; (**c**,**d**), *Eucera*; (**e**,**f**) *Hylaeus*; (**g**,**h**), *Apis mellifera*.

Following the Sanger sequencing of ArLV1-IL-Bh, the obtained complete genome sequence served as a reference template for the assembly of the HTS reads in order to re-assess the genome integrity of the plant comovirus in the bee samples. The analysis revealed a complete coverage of both RNA1 and RNA2 of ArLV1-IL-Bh in all analyzed bee populations (Figure 4). The highest depth in the reads, mapped to ArLV1-IL-Bh in *A. mellifera* compared to the wild bees (Figure 4g,h vs. Figure 4a–f), apparently reflected the high number of clean reads obtained from the honeybee sample compared to the wild bees while all bee samples showed a similar percentage of number of reads mapped to ArLV1-IL-Bh genome (Table 3).

### 3.6. Bee Tendency to Visit Brassica Flowers

**Figure 5:**
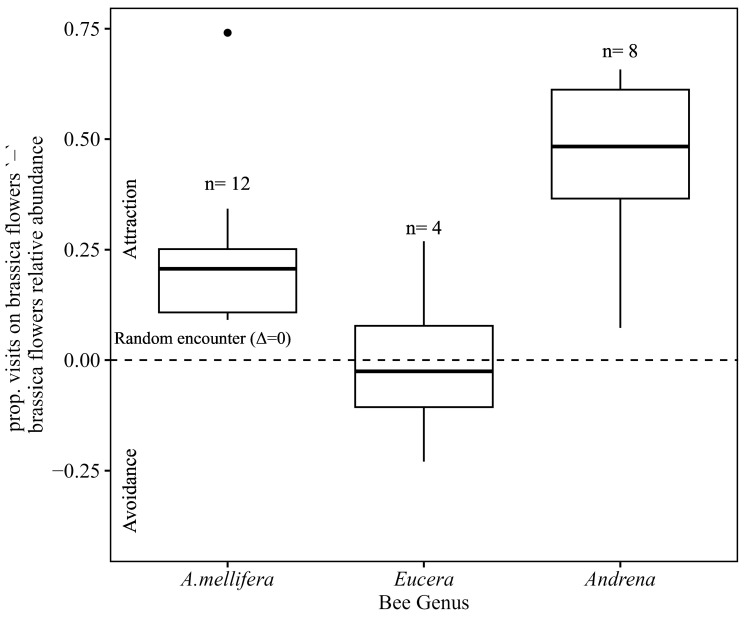
The distributions of differences between the proportion of bee visits on brassica flowers (crucifers) and the relative abundance of brassica plants on each sampling day. Positive and negative values indicate attraction to and avoidance of brassica plants, respectively. The dashed line represents visitation rates that are expected under random encounters. “●” a black dot represents an outlier value.

We asked whether the presence of ArLV1-IL-Bh in all bee samples was positively correlated with the bees’ tendency to visit brassica flowers. The median of the difference between the proportion of visits to brassica plants by each bee genus and the relative abundance of brassica plants in the floral community in the site was 0.2 (with an interquartile range (IQR) of 0.108–0.251) and 0.5 (IQR = 0.365–0.612) for honeybees (*A. mellifera*) and mining bees (*Andrena*), respectively (Figure 5). Both were significantly different from zero (Wilcoxon signed-rank test; *p* = 0.0004 and 0.007, respectively), indicating an attraction to brassica plants. Conversely, the median delta for long-horned bees (*Eucera*) was −0.02 (IQR = −0.106–0.077) and not significantly different from zero (Wilcoxon signed-rank test; *p* = 1) (Figure 5).

### 3.7. A Phylogenetic Tree Analysis

**Figure 6:**
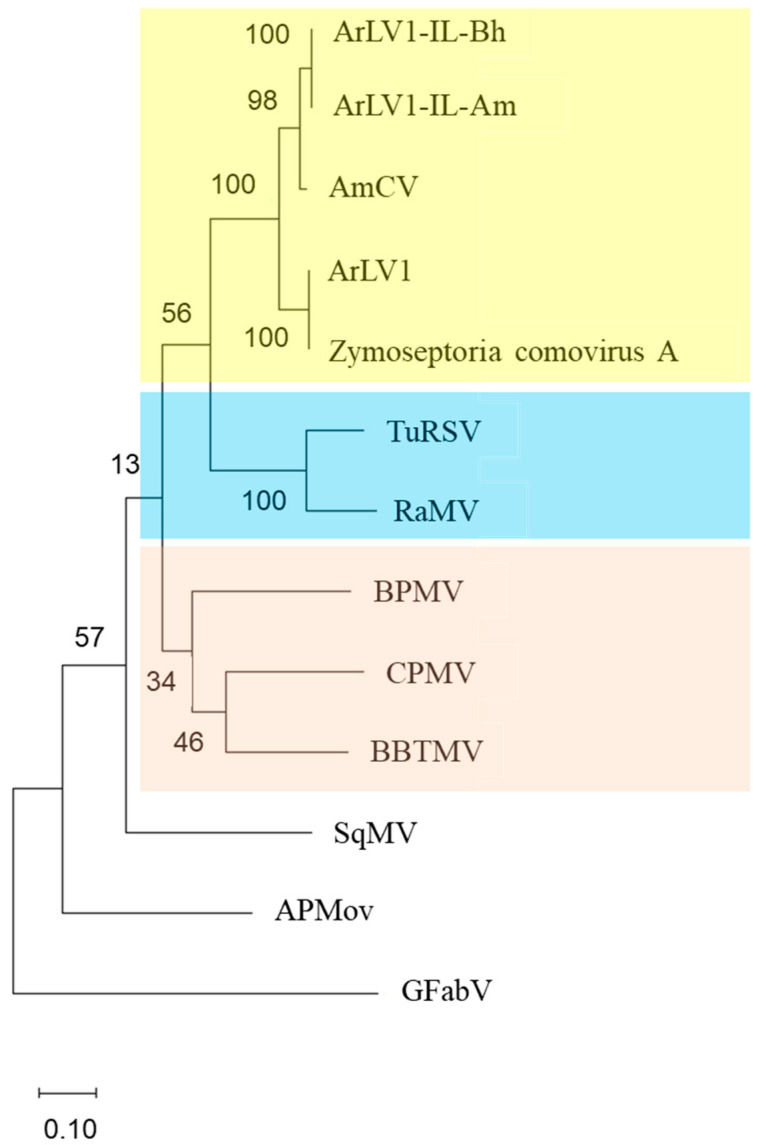
A phylogenetic tree based on *Comovirinae* subfamily Pro-Pol region. The following viruses and virus isolates, along with the ArLV1-IL-Bh (OR840696) and ArLV1-IL Am (OR840694), were included: Andean potato mottle virus (APMoV), MN148891; cowpea mosaic virus (CPMV), X00206; broad bean true mosaic virus (BBTMV), GU810903; bean pod mottle virus (BPMV), M62738; *Apis mellifera* associated comovirus (AmCV), OP972917; Arabidopsis latent virus 1 (ArLV1), MH899120; Zymoseptoria comovirus A, MK231051; turnip ringspot virus (TuRSV), GQ222381; radish mosaic virus (RaMV), AB295643; squash mosaic virus (SqMV), AB054688; and grapevine fabavirus (GFabV), KX241482.

A phylogenetic tree was constructed based on the alignment of amino acid sequences of the conserved Pro-Pol region. A high similarity was observed between the ArLV1-IL-Bh and the comovirus found in sampled honeybees ArLV1-IL-Am. Both ArLV1-IL isolates were clustered in a clade with AmCV, while ArLV1 and Zymoseptoria comovirus A sequences were clustered in the nearest separate clade. The Israeli strains ArLV1-IL-Bh and ArLV1-IL-Am were separated from AmCV in a sub-cluster. A cluster that included ArLV1-IL isolates, AmCV, ArLV1, and Zymoseptoria comovirus A was separated from other clusters, including TuRSV. The grapevine fabavirus (GFabV) served as an outgroup (Figure 6).

## 4. Discussion

We have identified a strain of comovirus in field-collected wild *B. hirta* plants. The characterization of the mustard comovirus strain showed high similarity to AmCV (accession numbers OP972917-20) and ArLV1 (accession numbers MH899120-21), the latter submitted to GenBank early in 2018; according to the ICTV taxonomy criteria, the isolate was designated as ArLV1-IL-Bh. The alignments of the putative amino acid sequence of ArLV1-IL-Bh at the Pro-Pol region showed a 96.5% and 90.3% similarity to AmCV and ArLV1, respectively. The putative amino acids of the CPs of ArLV1-IL-Bh shared a 93% and 82% similarity with AmCV and ArLV1, respectively. The putative cleavage sites of the 3C-like proteinase were identical between the ArLV1-IL-Bh and AmCV. However, when compared to ArLV1, the putative sites identified at the Pro-Co/Helicase domain differed. In ArLV1, the putative cleavage site was ^308^VAQ/AGP^313^, while in ArLV1-IL-Bh and AmCV, the cleavage site was ^308^VAQ/SGP^313^. Other putative 3C-like proteinase cleavage sites in the polyprotein of RNA1 and the small polyprotein of RNA2 were similar between ArLV1 and ArLV1-IL-Bh.

We have described unique symptoms of ArLV1-IL-Bh upon infection of the mustard plant *B. rapa* var. *perviridis*. Symptom manifestations of ringspot were associated with the infection. Ringspots are known characteristic symptoms of TuRSV and viruses belonging to the *Fabavirus* genus in the *Comovirinae* subfamily. Fabaviruses are transmitted by mechanical sap inoculations and aphids in a non-persistent manner and have a broad host range [2,51]. However, the phylogenetic tree analysis showed a separate clustering of ArLV1-IL isolates and TuRSV when grapevine fabavirus (GFabV) served as an outgroup (Figure 6). Host range analyses revealed that, similar to ArLV1, the *Brassicaceae* and *Solanaceae* family members were infected by the ArLV1-IL-Bh, and we added members of the *Amaranthaceae* and *Fabaceae* families to the ArLV1-IL-Bh host range (Table 2). 

There are several modes of comovirus transmission that were reported, including mechanical means [2], beetles [7], and seed transmission [8]. In the current study, we have shown that ArLV1-IL-Bh can be mechanically transmitted between plants, but furthermore, we have identified ArLV1-IL-Bh in managed honeybees and three wild bee genera, which are phylogenetically distant [24]. A whole-genome coverage of ArLV1-IL-Bh was demonstrated in all four bee libraries (Figure 4).

The reads mapped to ArLV1-IL-Bh encompassed both RNA1 and RNA2 in the four tested bee genera (Figure 4). Among the bees, the mapped reads showed a similar percentage of reads that were mapped to the ArLV1-IL-Bh reference genome, with 0.4–0.44% and 0.34–0.37% of the reads being mapped to RNA1 and RNA2, respectively (Table 3). The minor differences in the abundance of the two segments in the bee libraries (Figure 4, Table 3) were previously reported for numerous *A. thaliana* accessions hosting ArLV1 [8]. This phenomenon may result from separate encapsidations of the bipartite genome in the host plants, which are reported to be host-specific in other systems [52,53,54].

Low percentage values of the mapped reads are in accordance with a non-replicating virus in a non-host vector where the viral particles externally adhered to the bees [10,12,13]. Furthermore, the percentage of viral RNA reads did not seem to co-vary with the tendencies of the studied genera’s foraging behavior on brassica plants (Figure 5). The low virus loads of ArLV1-IL-Bh are in accordance with passive adherence via floral visitation, suggesting that the quantity of externally adhering virus particles saturates rapidly at lower visitation frequencies compared to the proportion of brassica flowers in the field. In addition, this study focused on bees that significantly differ in their morphological traits, including body size, tongue length, and relative hair cover density, as well as foraging behavior, different pollen collection mechanisms, nesting habits, and more [24]. These differences might affect the efficiency of plant virus vectoring by bees. These results could also suggest that the comovirus ArLV1-IL-Bh was shared between the tested genera in the specific geographic region, as was previously demonstrated in viruses infecting arthropods [22,23]. Alternatively, the shared source of ArLV1-IL-Bh acquisition might be a plant unrelated to the *Brassicaceae* family that hosts the comovirus. 

The managed and wild bees may be potential non-host vectors of ArLV1-IL-Bh and presumably ArLV1 in the wild, implying a role of bee populations in comovirus spread in wild vegetation, as suggested for AmCV in managed honeybees [20]. In addition, the seed transmission of ArLV1 [8] suggests viral presence in pollen grains. This transmission mode supports the possibility that bee pollinators would transmit the comoviruses by virus adherence to their body parts, as was observed with other mechanically transmitted viruses [10,12,13]. However, further studies are necessary to establish the transmission of ArLV1-IL-Bh via bee foraging behavior. A broad spectrum view of this possible transmission mode of ArLV1-IL-Bh opens a question regarding the significance of the possible contribution of the managed and wild bees to comovirus spread compared to the reported seed transmission of the virus [8].

It has been proposed that a low virulence could allow plant-pathogen mutualistic benefits by conferring tolerance toward abiotic stress or cross-protection against co-infecting pathogenic viruses [55,56,57]. This might be the case for ArLV1-IL-Bh, which caused distinct symptoms in the *B. rapa* var. *perviridis* mustard plants with no significant impact on the host plant. In the natural habitats, the *B. hirta* plants infected by ArLV1-IL-Bh harbored a dense population of the aphid *B. brassicae* but were asymptomatic. Whether ArLV1-IL-Bh protects brassica plants from aphid-transmitted viruses is unknown.

## 5. Conclusions

This study described a possible contribution of managed and wild pollinators to the long-term adaptation of plants with a seed-borne latent comovirus. Our results demonstrate the complexity of these interactions when multiple factors are involved in the plant virus’s natural spread. Modulating the characteristics of virus–host plant biological interactions could shed light on trade-offs for both plants and viruses in nature. 

## Figures and Tables

**Table 1:** Primers are used for sequencing and detection of the viral genome.

No. and Orientation	RNA Partite	Position (bp)	Sequence (5′–3′)
F1	II	1	TCCGCCAGTACTGGGGAG
F3	I	4634	GTGGAATACCTTCTGGATTTCC
F4	I	668	AAGCTATCGATTGGACAGTTG
F5	I	4223	GTCCAAAGGATGAAAAACTGC
F6	II	1948	TATCTATGACTATAGATTGGTTT
F7	II	461	AGCTCAAGCACTGCATTTGAA
F8	I	2104	GAATTTCATTCGTATGGTGAT
F9	I	4	GAACAGGACCAGGGTCCGC
F10	I	1092	AGCATTTGGTTGTCCCACTATCATTG
F11	I	2222	TCGATAAATTTGAGCATCTACTG
F14	II	689	TGGTGAAAATGAAGTGGTTCAC
F15	II	2120	AGGAGGCACTGGAGTAG
R1	I	748	TAGGGCAATATTTTTCAACCAC
R2	I	4062	GGGAAATCCTTCGGATGTG
R3	I	4598	AAGTCGGGAACAGCAAGCTA
R5	I	3231	ACAGAGCTCACTATTTTCAAAA
R7	I	1302	CTGCCAAACAAAATTTTGCAAGC
R8	I	2329	GAACAAATTGCGCCTCCTGT
R9	I	3520	CAACTTTAGCTACAACCAGAGA
R10	I	5921	GAAAATATCATAACGCGACATATAAC
R11	II	1287	TAGAACCAATGGCAGGAAGGT
R12	II	2168	AGAACTCAAAGCGTTAGGCA
R13	II	3445	ATGCGATATGATAAATCAAAATAC

**Table 2:** A host range analysis for ArLV1-IL-Bh.

Plant	Family	Symptoms	RT-PCR
*Brassica perviridis*	*Brassicaceae*	Ringspot	Positive
*B. rapa*	*Brassicaceae*	No symptoms	Negative
*Raphanus sativus*	*Brassicaceae*	No symptoms	Positive
*B. oleracea*	*Brassicaceae*	No symptoms	Positive
*Nicotiana benthamiana*	*Solanaceae*	Mosaic	Positive
*N. glutinosa*	*Solanaceae*	No symptoms	Negative
*Datura stramonium*	*Solanaceae*	No symptoms	Positive
*Vicia faba*	*Fabaceae*	No symptoms	Positive
*Vigna unguiculata*	*Fabaceae*	No symptoms	Positive
*Chenopodium amaranticolor*	*Amaranthacea*	No symptoms	Positive
*C. murale*	*Amaranthacea*	No symptoms	Positive
*C. quinoa*	*Amaranthacea*	No symptoms	Positive
*Gomphrena globosa*	*Amaranthacea*	No symptoms	Positive

**Table 3:** HTS read the results of four sampled bee populations.

Library	Total Number of Reads	Total Number of Reads after Cleaning	* Number of Reads Mapped to RNA1	* Number of Reads Mapped to RNA2
*Andrena*	18,749,336	18,740,291 (99.95%)	75,028 (0.40%)	63,370 (0.34%)
*Eucera*	27,055,141	27,039,024 (99.94%)	116,760 (0.43%)	98,010 (0.36%)
*Apis mellifera*	45,167,229	45,140,094 (99.94%)	199,587 (0.44%)	168,906 (0.37%)
*Hylaeus*	33,800,839	33,784,842 (99.95%)	139,155 (0.41%)	115,759 (0.34%)

* Assembled on ArLV1-IL-Bh.

**Table 4:** BLAST analysis of HTS de novo assembled contigs derived from managed and wild bees’ pooled samples.

Honey bee (*Apis mellifera*)									
Contig	RNA	Position (nt)	Contig Size (bp)	* Mismatch	* INDELs	% ArLV1-IL-Bh aa	% ArLV1 aa	% Zymoseptoria Comovirus A aa	% AmCV aa
2688	1	124–492	368	7	0	99	82	82	98
3388	1	1327–1647	320	3	0	100	89	89	98
2241	1	1645–4430	2785	19	0	99	90	90	97
39_1	1	4362–5907	1545	26	0	99	83	83	94
39_3	2	1–3378	3378	56	4	99.8	79.4	79.9	92.19
**Mining bee (*Andrena*)**									
3396	1	2715–3376	661	6	0	99.5	90.4	90.4	97.2
2094	1	3361–3624	263	2	0	100	89.7	89.7	98.8
3946	1	3642–4061	419	4	0	100	87.1	87.1	96.4
67_2	1	4164–5892	1728	30	1	99.6	85.2	85.2	94.6
2253	2	182–744	562	5	0	99	67	67	86
1218	2	959–1199	240	4	0	79	76	76	79
4073	2	1184–1501	317	6	0	99	66	68	90
67_1	2	1563–3443	1880	25	3	99	82	82	93
**Long-horned bee (*Eucera*)**									
4418	1	4559–4820	261	7	0	99	92	92	98
2837	1	5051–5784	733	9	0	99	78	78	92
3346	2	498–744	246	2	0	99	92	93	95
2617	2	1057–1328	271	4	0	100	90	91	98
2924_1	2	1429–1634	205	5	0	97	53	77	90
2924_0	2	1612–2155	543	14	0	74	68	69	71
1924	2	2313–2926	613	9	0	97	79	79	95
4230	2	2912–3317	405	6	2	100	66	66	84
**Masked bee (*Hylaeus*)**									
7428	1	3531–3924	393	6	0	85	73	73	80
9174	1	4032–4347	315	4	0	99	83	83	94
12,606	1	4704–4973	269	10	0	99	96	96	96
5150	1	5054–5597	543	7	0	100	90	90	99
12,033	2	822–1117	295	13	0	99	98	98	99
12,764	2	1548–1818	270	8	0	98	87	88	91
1842	2	1970–2208	238	2	0	100	86	86	94
4413	2	2193–3254	1061	23	1	98	78	89	93

* Mismatch and INDELs were determined on ArLV1-IL-Bh genome reference. ArLV1-IL-Bh, (OR840696, OR840697); ArLV1, (MH899120, MH899121); Zymoseptoria comovirus A (MK231051, MK231039); AmCV (OP972917-20); aa, denoted for amino acids.

## Data Availability

The data are available in the manuscript. The two ArLV1-IL isolates were deposited in the GenBank.

## Supplementary Materials

The following supporting information can be downloaded at: https://www.mdpi.com/article/10.3390/plants13050671/s1, Table S1: Wild bee sampling conducted between February and May 2021 at five shrubland sites in the Judean foothills, central Israel.

## References

[1] van Kammen A. (1972). Plant viruses with a divided genome. Annu. Rev. Phytopathol..

[2] Thompson J.R., Dasgupta I., Fuchs M., Iwanami T., Karasev A.V., Petrzik K., Sanfaçon H., Tzanetakis I., van der Vlugt R., Wetzel T. (2017). ICTV virus taxonomy profile: Secoviridae. J. Gen. Virol..

[3] Lomonossoff G., Shanks M. (1983). The nucleotide sequence of cowpea mosaic virus B RNA. EMBO J..

[4] van Wezenbeek P., Verver J., Harmsen J., Vos P., van Kammen A. (1983). Primary structure and gene organization of the middle-component RNA of cowpea mosaic virus. EMBO J..

[5] Peters S., Voorhorst W., Wery J., Wellink J., van Kammen A. (1992). A regulatory role for the 32K protein in proteolytic processing of cowpea mosaic virus polyproteins. Virology.

[6] Wellink J., Van Kammen A. (1989). Cell-to-cell transport of cowpea mosaic virus requires both the 58K/48K proteins and the capsid proteins. J. Gen. Virol..

[7] Fulton J.P., Gergerich R.C., Scott H.A. (1987). Beetle Transmission of Plant Viruses. Annu. Rev. Phytopathol..

[8] Verhoeven A., Kloth K.J., Kupczok A., Oymans G.H., Damen J., Rijnsburger K., Jiang Z., Deelen C., Sasidharan R., van Zanten M. (2023). Arabidopsis latent virus 1, a comovirus widely spread in *Arabidopsis thaliana* collections. New Phytol..

[9] Bristow P.R., Martin R.R. (1999). Transmission and the role of honeybees in field spread of blueberry shock ilarvirus, a pollen-borne virus of highbush blueberry. Phytopathology.

[10] Okada K., Kusakari S.-I., Kawaratani M., Negoro J.-I., Ohki S.T., Osaki T. (2000). Tobacco mosaic virus is transmissible from tomato to tomato by pollinating bumblebees. J. Gen. Plant Pathol..

[11] Shipp J., Buitenhuis R., Stobbs L., Wang K., Kim W., Ferguson G. (2008). Vectoring of *Pepino mosaic virus* by bumble-bees in tomato greenhouses. Ann. Appl. Biol..

[12] Darzi E., Smith E., Shargil D., Lachman O., Ganot L., Dombrovsky A. (2018). The honeybee *Apis mellifera* contributes to *Cucumber green mottle mosaic virus* spread via pollination. Plant Pathol..

[13] Levitzky N., Smith E., Lachman O., Luria N., Mizrahi Y., Bakelman H., Sela N., Laskar O., Milrot E., Dombrovsky A. (2019). The bumblebee Bombus terrestris carries a primary inoculum of Tomato brown rugose fruit virus contributing to disease spread in tomatoes. PLoS ONE.

[14] Cunningham M.M., Tran L., McKee C.G., Polo R.O., Newman T., Lansing L., Griffiths J.S., Bilodeau G.J., Rott M., Guarna M.M. (2022). Honey bees as biomonitors of environmental contaminants, pathogens, and climate change. Ecol. Indic..

[15] Fetters A.M., Ashman T. (2023). The pollen virome: A review of pollen-associated viruses and consequences for plants and their interactions with pollinators. Am. J. Bot..

[16] Granberg F., Vicente-Rubiano M., Rubio-Guerri C., Karlsson O.E., Kukielka D., Belák S., Sánchez-Vizcaíno J.M. (2013). Metagenomic detection of viral pathogens in Spanish honeybees: Co-infection by aphid lethal paralysis, Israel acute paralysis and Lake Sinai viruses. PLoS ONE.

[17] Schoonvaere K., De Smet L., Smagghe G., Vierstraete A., Braeckman B.P., de Graaf D.C. (2016). Unbiased RNA shotgun metagenomics in social and solitary wild bees detects associations with eukaryote parasites and new viruses. PLoS ONE.

[18] Galbraith D.A., Fuller Z.L., Ray A.M., Brockmann A., Frazier M., Gikungu M.W., Martinez J.F.I., Kapheim K.M., Kerby J.T., Kocher S.D. (2018). Investigating the viral ecology of global bee communities with high-throughput metagenomics. Sci. Rep..

[19] Roberts J.M.K., Ireland K.B., Tay W.T., Paini D. (2018). Honey bee-assisted surveillance for early plant virus detection. Ann. Appl. Biol..

[20] Kwon M., Jung C., Kil E.-J. (2023). Metagenomic analysis of viromes in honey bee colonies (*Apis mellifera*; Hymenoptera: Apidae) after mass disappearance in Korea. Front. Cell. Infect. Microbiol..

[21] Lee E., Vansia R., Phelan J., Lofano A., Smith A., Wang A., Bilodeau G.J., Pernal S.F., Guarna M.M., Rott M. (2023). Area Wide Monitoring of Plant and Honey Bee (*Apis mellifera*) Viruses in Blueberry (*Vaccinium corymbosum*) Agroecosystems Facilitated by Honey Bee Pollination. Viruses.

[22] Levitt A.L., Singh R., Cox-Foster D.L., Rajotte E., Hoover K., Ostiguy N., Holmes E.C. (2013). Cross-species transmission of honey bee viruses in associated arthropods. Virus Res..

[23] Furst M.A., McMahon D.P., Osborne J.L., Paxton R.J., Brown M.J.F. (2014). Disease associations between honeybees and bumblebees as a threat to wild pollinators. Nature.

[24] Michener C.D. (2000). The Bees of the World.

[25] Wood T.J., Roberts S.P. (2017). An assessment of historical and contemporary diet breadth in polylectic Andrena bee species. Biol. Conserv..

[26] Hennessy G., Goulson D., Ratnieks F.L.W. (2020). Population assessment and foraging ecology of nest aggregations of the rare solitary bee, Eucera longicornis at Gatwick Airport, and implications for their management. J. Insect Conserv..

[27] Müller A. (2023). The hidden diet—Examination of crop content reveals distinct patterns of pollen host use by Central European bees of the genus Hylaeus (Hymenoptera, Colletidae). Alp. Èntomol..

[28] Gathmann A., Tscharntke T. (2002). Foraging ranges of solitary bees. J. Anim. Ecol..

[29] Zurbuchen A., Landert L., Klaiber J., Müller A., Hein S., Dorn S. (2010). Maximum foraging ranges in solitary bees: Only few individuals have the capability to cover long foraging distances. Biol. Conserv..

[30] Hibi T., Furuki I. (1985). Melon Necrotic Spot Virus.

[31] Dombrovsky A., Pearlsman M., Lachman O., Antignus Y. (2009). Characterization of a new strain of Eggplant mottled crinkle virus (EMCV) infecting eggplants in Israel. Phytoparasitica.

[32] Rosner A., Ginzburg I., Bar-Joseph M. (1983). Molecular cloning of complementary DNA sequences of citrus tristeza virus RNA. J. Gen. Virol..

[33] Khandekar S., He J., Leisner S. (2009). Complete nucleotide sequence of the Toledo isolate of turnip ringspot virus. Arch. Virol..

[34] Laemmli U.K. (1970). Cleavage of structural proteins during the assembly of the head of bacteriophage T4. Nature.

[35] Bolger A.M., Lohse M., Usadel B. (2014). Trimmomatic: A flexible trimmer for Illumina sequence data. Bioinformatics.

[36] Zheng Y., Gao S., Padmanabhan C., Li R., Galvez M., Gutierrez D., Fuentes S., Ling K.-S., Kreuze J., Fei Z. (2017). VirusDetect: An automated pipeline for efficient virus discovery using deep sequencing of small RNAs. Virology.

[37] Zerbino D.R. (2010). Using the velvet de novo assembler for short-read sequencing technologies. Curr. Protoc. Bioinform..

[38] Houtgast E.J., Sima V.-M., Bertels K., Al-Ars Z. (2018). Hardware acceleration of BWA-MEM genomic short read mapping for longer read lengths. Comput. Biol. Chem..

[39] Grabherr M.G., Haas B.J., Yassour M., Levin J.Z., Thompson D.A., Amit I., Adiconis X., Fan L., Raychowdhury R., Zeng Q. (2011). Trinity: Reconstructing a full-length transcriptome without a genome from RNA-Seq data. Nat. Biotechnol..

[40] Buchfink B., Xie C., Huson D.H. (2015). Fast and sensitive protein alignment using DIAMOND. Nat. Methods.

[41] Langmead B., Salzberg S.L. (2012). Fast gapped-read alignment with Bowtie 2. Nat. Methods.

[42] Danecek P., Bonfield J.K., Liddle J., Marshall J., Ohan V., Pollard M.O., Whitwham A., Keane T., McCarthy S.A., Davies R.M. (2021). Twelve years of SAMtools and BCFtools. GigaScience.

[43] Sanfaçon H., Wellink J., Le Gall O., Karasev A., van der Vlugt R., Wetzel T. (2009). Secoviridae: A proposed family of plant viruses within the order Picornavirales that combines the families Sequiviridae and Comoviridae, the unassigned genera Cheravirus and Sadwavirus, and the proposed genus Torradovirus. Arch. Virol..

[44] Ritzenthaler C., Viry M., Pinck M., Margis R., Fuchs M., Pinck L. (1991). Complete nucleotide sequence and genetic organization of grapevine fanleaf nepovirus RNA1. J. Gen. Virol..

[45] Gorbalenya A.E., Blinov V.M., Donchenko A.P., Koonin E.V. (1989). An NTP-binding motif is the most conserved sequence in a highly diverged monophyletic group of proteins involved in positive strand RNA viral replication. J. Mol. Evol..

[46] Mayoa M., Fritsch C. (1994). A possible consensus sequence for VPg of viruses in the family *Comoviridae*. FEBS Lett..

[47] Bazan J.F., Fletterick R.J. (1988). Viral cysteine proteases are homologous to the trypsin-like family of serine proteases: Structural and functional implications. Proc. Natl. Acad. Sci. USA.

[48] Gorbalenya A.E., Donchenko A.P., Blinov V.M., Koonin E.V. (1989). Cysteine proteases of positive strand RNA viruses and chymotrypsin-like serine proteases: A distinct protein superfamily with a common structural fold. FEBS Lett..

[49] Poch O., Sauvaget I., Delarue M., Tordo N. (1989). Identification of four conserved motifs among the RNA-dependent polymerase encoding elements. EMBO J..

[50] Koonin E.V., Mushegian A.R., Ryabov E.V., Dolja V.V. (1991). Diverse groups of plant RNA and DNA viruses share related movement proteins that may possess chaperone-like activity. J. Gen. Virol..

[51] Kobayashi Y.O., Kobayashi A.K.Y.O., Hagiwara K., Uga H., Mikoshiba Y., Naito T., Honda Y., Omura T. (2005). Gentian mosaic virus: A new species in the genus *Fabavirus*. Phytopathology.

[52] Sainsbury F., Cañizares M.C., Lomonossoff G.P. (2010). *Cowpea mosaic* virus: The plant virus–based biotechnology workhorse. Annu. Rev. Phytopathol..

[53] Sicard A., Yvon M., Timchenko T., Gronenborn B., Michalakis Y., Gutierrez S., Blanc S. (2013). Gene copy number is differentially regulated in a multipartite virus. Nat. Commun..

[54] Moreau Y., Gil P., Exbrayat A., Rakotoarivony I., Bréard E., Sailleau C., Viarouge C., Zientara S., Savini G., Goffredo M. (2020). The genome segments of bluetongue virus differ in copy number in a host-specific manner. J. Virol..

[55] Roossinck M.J. (2015). A new look at plant viruses and their potential beneficial roles in crops. Mol. Plant Pathol..

[56] Aguilar E., Lozano-Duran R. (2022). Plant viruses as probes to engineer tolerance to abiotic stress in crops. Stress Biol..

[57] Sáez C., Pagán I. (2023). When plants are Trojan horses for viruses. New Phytol..

