# Supplementary material for: A Distinct Arabidopsis Latent Virus 1 Isolate Was Found in Wild Brassica hirta Plants and Bees, Suggesting the Potential Involvement of Pollinators in Virus Spread
Source: Plants (Basel). 2024 Feb 28;13(5):671. doi: 10.3390/plants13050671 (PMC10935448; doi:10.3390/plants13050671)
Supplement: Supplementary file 1 [file plants-13-00671-s001.zip › plants-2844729-supplementary.pdf]

Supplementary Table S1. Wild bee sampling conducted between February and May 2021 at five shrubland sites in the Judean foothills, central Israel.

**Site name**	**Coordinates**	**Survey's dates** (*wild bee sampling [An/Eu/Hyl])
Agur	31.6937, 34.9182	21/2[An]; 28/2 [An]; 1st (7/3) [An]; 2nd (14/3) [An]; 3rd (21+23/3)[Eu]; 4th (1/4) [An]; 5th (14/4) [An]; 6th (28/4) [An,Hyl]
Lachish	31.5913, 34.837	1st (8/3) [An,Eu]; 2nd (15/3) [An,Eu]; 3rd (22/3) [An,Eu];4th (4/4) [Eu]; 5th (13/4) [An];6th (22/4) [An,Hyl];7th (12/5)[Hyl]
Luzit	31.6793, 34.8887	1st (9/3)[Eu];2nd (16/3) [An, Eu];3rd (31/3) [Eu];4th (5/4) [An, Eu];5th (20/4) [Eu];6th (29/4) [Eu,Hyl];7th (5/5)
Galon	31.6305, 34.8437	1st (10/3) [An];2nd (17/3) [Eu An];3rd (25/3) [An, Eu];4th (7/4) [An];5th (25/4) [An,Hyl];6th (2/5) [Hyl];7th (6/5) [Hyl]
Tarum	31.7975, 34.965	1st (11/3) [An];2nd (18/3) [An];3rd (30/3) [An];4th (8/4) [An];5th (23/4);6th (3/5);7th (11/5)

*An, solitary mining bees (*Andrena*); Eu, long-horned bees (*Eucera*); Hyl, masked bees (*Hylaeus*)

Apis mellifera was sampled on each of the collection days.
